# Evaluation of Z-plasty versus Heineke-Mikulicz scrotoplasty in the management of penoscrotal web in pediatric age group

**DOI:** 10.1186/s12894-024-01450-7

**Published:** 2024-03-22

**Authors:** Ahmed Elrouby

**Affiliations:** Department of Pediatric Surgery, Elshatby University Hospital, Faculty of Medicine, Alexandria University, Alexandria, Egypt

**Keywords:** Penoscrotal web, Z-scrotoplasty, Heineke-Mikulicz, Scrotoplasty

## Abstract

**Background:**

The penoscrotal web may be congenital or acquired following excessive ventral skin removal during circumcision. Several surgical techniques were described for the treatment of congenital webbed penis without a clear comparison between their outcomes. This prospective study aimed at comparing the surgical results of Z-scrotoplasty and Heineke-Mikulicz scrotoplasty in the treatment of congenital webbed penis in uncircumcised pediatric patients.

**Methods:**

Our study included 40 uncircumcised patients who were divided randomly into two groups; Group A included 20 patients who were treated by Z-scrotoplasty and Group B included the other 20 patients who were treated by Heineke-Mikulicz scrotoplasty. All patients were circumcised at the end of the procedure.

**Results:**

The surgical outcome was good without a significant difference between the two groups in 36 patients. Recurrent webbing developed in one patient of Group A and in three patients of Group B (^FE^ *p* = 0.605) The only significant difference between the two groups was the operative duration which was shorter in Group B than in Group A (*P* < 0.001*).

**Conclusions:**

Treatment of congenital penoscrotal web in the pediatric age group could be done with either Z-scrotoplasty or Heineke-Mikulicz scrotoplasty with satisfactory results, however, without significant difference in the surgical outcomes.

**Trial registration:**

• Registration Number: ClinicalTrials.gov ID: NCT05817760.

• Registration release date: April 5, 2023.

## Background

The webbed penis is a congenital condition in which a skin fold tethers the scrotum to the ventral penile shaft obscuring the penoscrotal angle. This anomaly is usually discovered in infancy or at circumcision. This anomaly usually leads to penile shortening and is considered a common cause of delayed circumcision [1].

Circumcision in the case of a webbed penis without releasing this web results in the downward urinary stream during childhood and makes future sexual function difficult during adulthood so circumcision without excision of the web is usually contraindicated and web correction is mandatory [2].

The main target of the treatment of penoscrotal web is to incise the web with ventral penile skin lengthening; this is conventionally done by transversely incising that web with vertical closure (Heineke-Mikulicz incision) [3].

Other innovations including Z-plasty, lateral para-penile incision, and other flap methods, like preputial skin flap rotation, etc., have been also described for the treatment of such conditions [4].

The Heineke-Mikulicz scrotoplasty is the most commonly used method for such conditions in the form of longitudinal incision and transverse closure. History of this technique belongs to Heineke who performed pyloroplasty for the first time for a patient presented with an obstructing pyloric mass in 1886. One year later in1887, Mikulicz described the same technique but for treatment of a bleeding peptic ulcer [5].

It was also described by Emmanuel Lee in 1976 in the treatment of intestinal strictures following Crohn’s disease [6].

RN Katariya et al. reported the usage of the same technique in the management of terminal ileal strictures [7].

This procedure was then widely used by many surgeons for variable conditions in which there is narrowing or stenosis to provide an additional length and/or width to a luminal structure. Its usage in the penoscrotal web involves transverse incision centered on the expected point of release of the web followed by longitudinal closure [1].

### Aim of the work

This study aimed to compare the surgical outcome of Z-scrotoplasty versus Heineke-Mikulicz scrotoplasty in the management of congenital penoscrotal web in the pediatric age group.

### Patients & methods

**Fig. 1:**
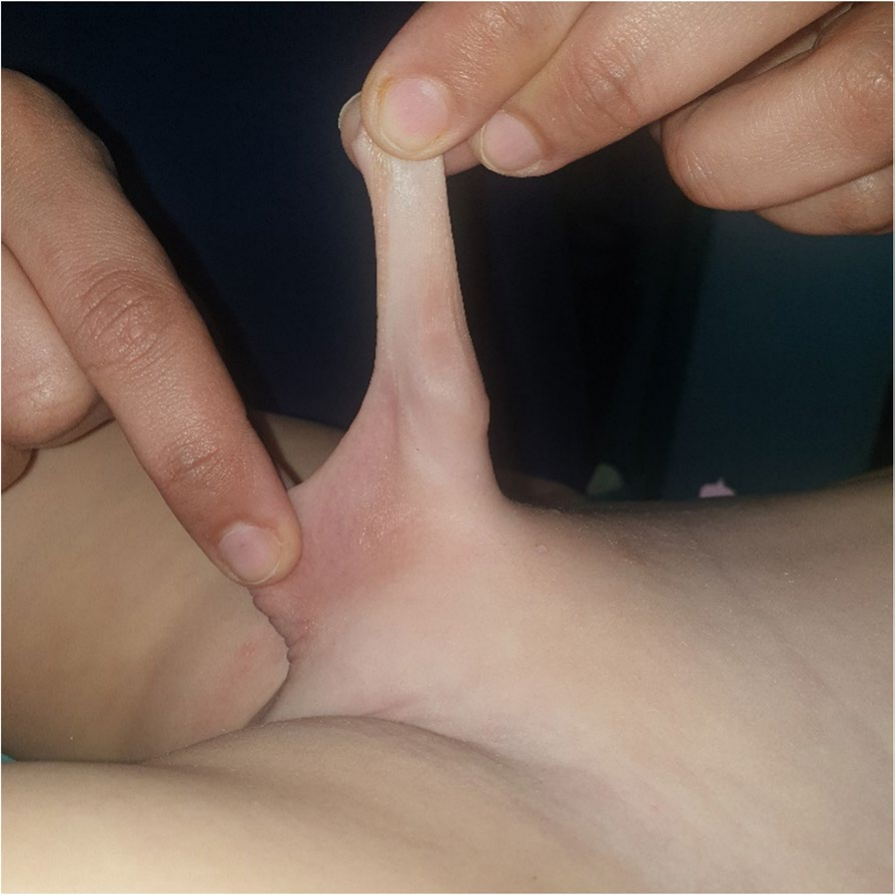
Congenital penoscrotal web

This prospective randomized interventional study was conducted on 40 patients having congenital penoscrotal web who were presented to Elshatby University Hospital from January 2019 to January 2021. Patients with any other congenital penile anomalies were excluded from our study (Fig. 1).

The age and weight at operation as well as the main complaint of parents or caregivers were recorded. The main complaint was an apparent small size penis, penoscrotal webbing, or a postponed circumcision by another surgeon due to the presence of the web. Patients with hypospadias, circumcised patients, micropenis, and/or torsion were excluded from this study. The studied patients were divided randomly into two groups; Group A included 20 patients who were treated by Z- scrotoplasty and Group B included another 20 patients who were treated by Heineke-Mikulicz scrotoplasty.

**Fig. 2:**
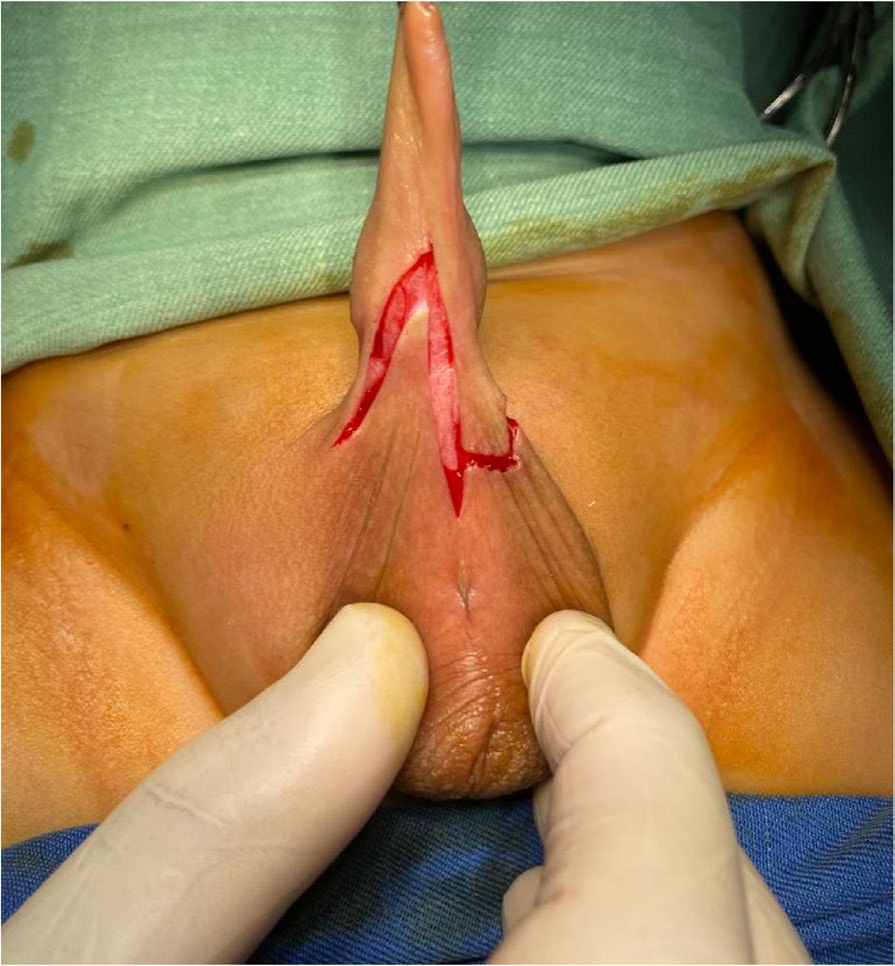
The Z- shape incision in patients of group A

Pre-operative investigations included the routine laboratory tests; PT, PTT, INR, BT, CT & CBC. Operative intervention was conducted under general anesthesia with skin preparation using povidone-iodine. The surgical procedure started in patients of Group A with the creation of a Z-shape incision with its longitudinal arm extending along the web and its two lateral limbs extending alongside the web (Fig. 2).

**Fig. 3:**
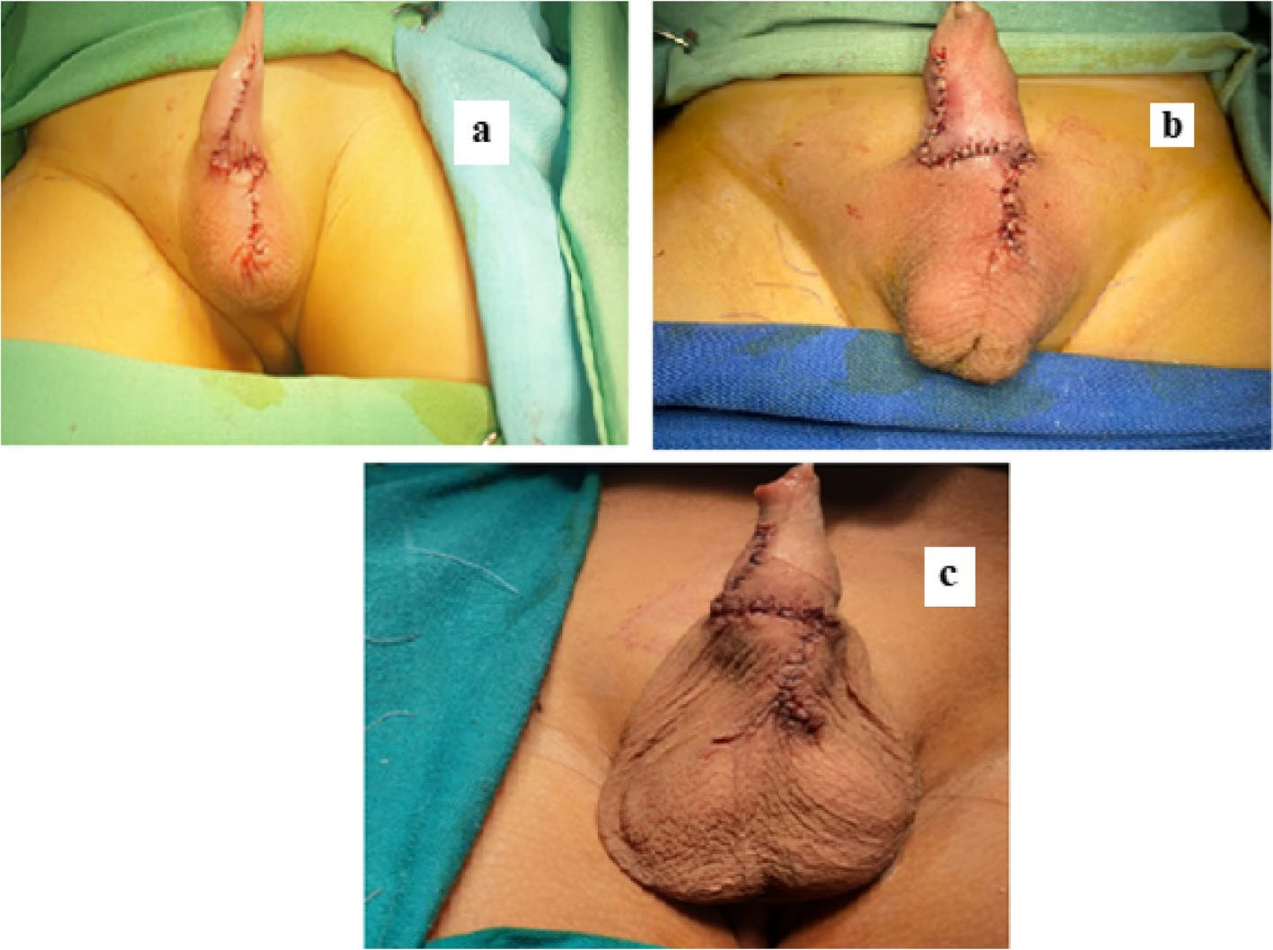
**a**, **b** & **c** Ventral skin closure in patients of Group A at the end of the operation (Z—scrotoplasty)

Complete and meticulous lateral dissection of the two flaps was done at a sufficient depth keeping skin vascularity. Simple closure of the skin flaps was done using Vicryl 6/0 after point-to-point hemostasis using bipolar diathermy (Fig. 3).

**Fig. 4:**
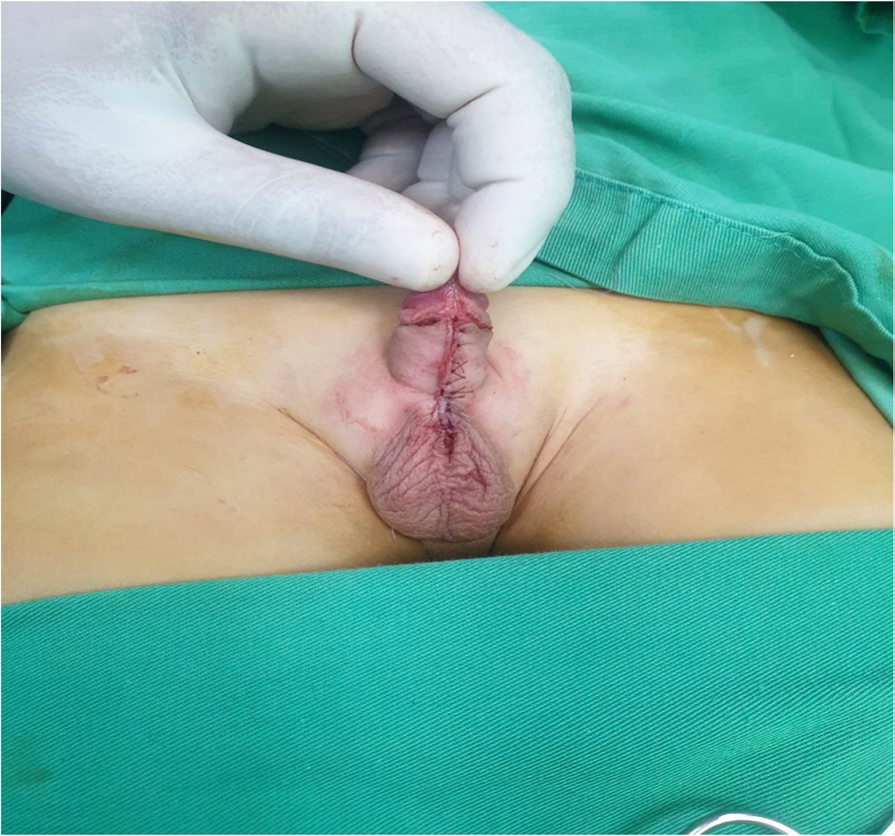
Ventral midline closure of a patient in group B at the end of the operation (Heineke-Mikulicz scrotoplasty)

The incision in patients of Group B was done transversely across the web at the level of the penoscrotal junction. Meticulous proximal and distal dissection of skin flaps was done preserving skin vascularity and allowing for tension-free vertical closure. Good hemostasis was done followed by longitudinal midline simple closure using the same suture material (Fig. 4).

**Fig. 5:**
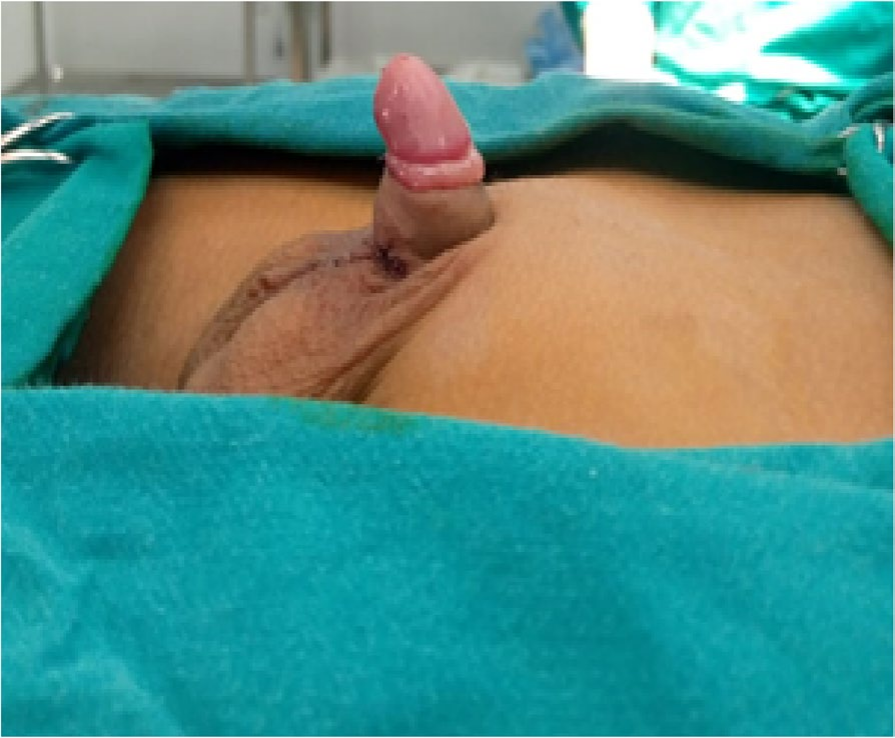
Circumcision with subcuticular closure of the mucocutaneous junction at the end of the procedure

The dartos layer was dissected in the two procedures from the penoscrotal angle without the need for its excision and circumcision was done in all patients with closure of the mucocutaneous junction with the same suture material in a subcuticular fashion at the end of the procedure (Fig. 5).

Follow-up was carried out at the end of the 1st postoperative week as well as at the 3rd, 6th, and 12th postoperative months during the regular visits at Elshatby University Hospital. The follow-up parameters included penile edema, hematoma, gangrene, recurrent webbing, and/or ventral curvature.

My study adheres to CONSORT guidelines and a checklist will be uploaded as an additional file during submission.

### Statistical analysis

The IBM SPSS software package version 20.0 (Armonk, NY: IBM Corp) was used to analyze our data describing the qualitative data as numbers and percentages. On the other hand, the quantitative data were described as range (minimum and maximum), mean, standard deviation, and median (IQR). The comparison between the two groups was done by using the Chi-square test for categorical variables, Fisher’s Exact or Monte Carlo correction for chi-square when more than 20% of the cells have an expected count less than 5 and Mann Whitney test for abnormally distributed quantitative variables. The level of significance was tested at the 5% level [8].

## Results

This study included 40 patients in the pediatric age group with their ages at operation ranging between 6 months and 6 years with an average of 29.9 ± 17.45 months. Patients of Group A were younger than patients of Group B without showing statistical significance (Table 1: *P* = 0.192).

**Table 1:** Difference between the two studied groups according to demographic data and operative duration

		**Group A** ***N*** ** = 20**	**Group B** ***N*** ** = 20**	**Test of significance**	***P***
**Age at operation** **(month)**	Min–Max	6.0 – 72.0	12.0 – 60.0	U = 151.50	0.192
	Mean ± SD	28.0 ± 21.77	31.80 ± 12.63		
	Median (IQR)	18.0 (12.0 – 48.0)	30.0 (24.0 – 36.0)		
**Weight at operation** **(Kg)**	Min–Max	8.0 – 30.0	13.0 – 35.0	U = 139.50	0.102
	Mean ± SD	17.70 ± 7.22	20.40 ± 5.56		
	Median (IQR)	16.0 (11.5 – 25.0)	18.50 (17.5 – 23.0)		
**Operative duration** **(min)**	Min – Max	35.0 – 60.0	20.0 – 40.0	U = 4.0^*^	< 0.001^*^
	Mean ± SD	45.50 ± 6.67	22.90 ± 4.58		
	Median (IQR)	45.0 (40.0 – 47.5)	22.0 (20.0 – 25.0)		

The patients’ body weight ranged between 8 and 35 kg with an average of 19.05 ± 6.42 kg. The measured body weight at operation was lower in patients of Group A than in patients of Group B without showing statistical significance (Table 1: *P* = 0.102).

The operative duration from skin incision to skin closure was measured and ranged between 20 and 60 min with an average of 34.2 ± 12.6 min. It took a statistically significantly shorter duration to operate on patients of Group B than in patients of Group A (Table 1: *P* < 0.001*).

The early follow-up of the studied patients at the end of the 1st post-operative week revealed the development of penile edema in 5 patients; 4 belonged to Group A and the other patient belonged to Group B; this difference did not show statistical significance. Conservative measures including warm bathes and oral anti-inflammatory drugs were sufficient for the management of such conditions (Table 1: ^FE^ *p* = 0.342).

Two patients developed postoperative subcutaneous hematoma; one in each group. Warm compresses were efficient for relieving this condition during the six post-operative weeks (Table 2: ^FE^ *p* = 1.000).

**Table 2:** Comparison of the two groups according to the postoperative follow-up parameters

	**Group A** ***N*** ** = 20**	**Group B** ***N*** ** = 20**	**Test of significance**	***P***
Edema	4 (20%)	1 (5%)	χ^2^ = 2.057	^FE^ *p* = 0.342
Hematoma	1 (5%)	1 (5%)	χ^2^ = 0.000	^FE^ *p* = 1.000
Skin coverage at the end of the operation	20 (100%)	19 (95%)	χ^2^ = 1.026	^FE^ *p* = 1.000
Recurrence & and wound contracture	1 (5%)	3 (15%)	χ^2^ = 1.111	^FE^ *p* = 0.605

Regarding penile skin coverage at the end of the operation; all of the studied patients had adequate ventral skin closure without significant difference between the two groups. However, one patient belonging to Group B showed tension at the level of the dorsal penoscrotal junction after ventral skin closure, so a dorsal 4 mm longitudinal midline release incision was done which relieved this tension adequately (Table 2: ^FE^ *p* = 1.000).

**Fig. 6:**
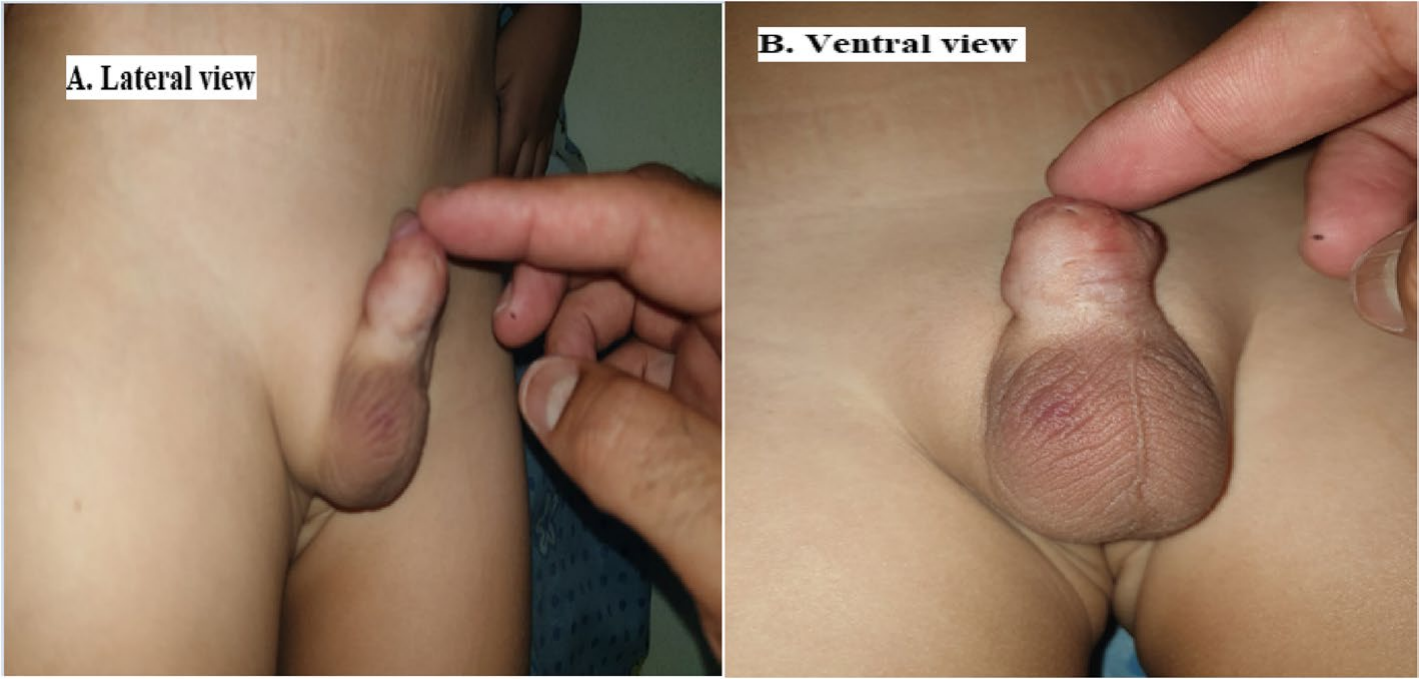
Post-operative follow-up of a patient of Group A (**A** Lateral view, **B** Ventral view)

Successful repair with good surgical outcomes together with smooth wound healing was achieved in 36 patients (90%) during the regular follow-up visits of the studied patients (Fig. 6).

The overall difference in the rate of postoperative complications between the two studied groups did not show statistical significance. Four patients developed post-operative wound contracture and recurrence of penile webbing; one patient in Group A developed mild acceptable curvature without the need for any surgical intervention and the other three patients in Group B developed severe wound contracture with evident recurrent webbing so a redo repair with Z-plasty was done for their management. The difference in the development of postoperative wound contracture and recurrent webbing between the two groups was not statistically significant (Table 2: ^FE^ *p* = 0.605).

One patient belonging to Group A showed a post-operative buried penis and managed conservatively with regular penile skin retraction and cleaning. Otherwise the final cosmetic result was acceptable among the studied patients as recorded by their parents.

## Discussion

The actual etiology of congenital webbed penis is not well known. However, one theory postulated that a congenital deficiency of the development of ventral penile skin may result in its shortage and consequently borrowing in scrotal tissue with a resulting penoscrotal web [9].

Although it may not cause any problem during childhood period except for abnormal urine stream; a congenital webbed penis can cause painful erections as well as making sexual intercourse challenging; this makes its correction mandatory [10].

Different surgical techniques were described by many surgeons for the correction of webbed penis. However, the comparison between these techniques was not described widely in the literature. R P Bonitz et al. compared three surgical techniques treating this condition namely; Heineke-Mikulicz (HM) scrotoplasty, VY scrotoplasty, and Z scrotoplasty without any difference as regards the follow-up results, and documented that all of such techniques could be safely used and the choice between them depends only on surgeon’s preferences [1].

M Maizels et al. reported in their study that circumcision of a straight webbed penis with a slanting up circumcision line leaving slightly more ventral skin to compensate for the web with regular post-operative push down of penile skin for regular cleaning could be efficient. However, the need for this regular procedure may limit the use of this technique and raise the importance of its correction [2].

Our study compared the outcomes of two surgical techniques for the correction of penoscrotal web namely Z-scrotoplasty and Heineke-Mikulicz scrotoplasty. The success rate in patients who were treated by Z-scrotoplasty was 95% with only one patient who developed postoperative wound contracture and recurrent webbing. On the other hand, the success rate in the case of Heineke-Mikulicz scrotoplasty was 85% with recurrent webbing in 3 patients in this group. However, this difference in the success rate was not statistically significant. This is similar to the results of a study conducted in 2020 for the management of webbed penis in circumcised patients which revealed that using either a Heineke-Mikulicz incision or multiple Z-plasty techniques results in favorable outcome without significant difference [3].

Our study distributed the included forty patients randomly between the two studied groups; twenty in each group without significant difference in the success rate in relation to the degree of web. On the other hand, El-Koutby M. in his series proposed a new classification of penoscrotal web and recommended the interventional technique according to the degree of the web to achieve the best result with the most acceptable cosmetic appearance [10].

R.P. Bonitz et al. described in their series that although Heineke Miculcz scrotoplasty is easier than Z- scrotoplasty; severe cases of penoscrotal web necissates the utilization of Z-scrotoplasty as recommended by El-Koutby M. Besides they discovered no significant difference as regards the surgical outcome as concluded in our study [1, 10].

This study reported a significantly shorter operative duration in patients who were treated by Heineke-Mikulicz scrotoplasty (22.90 min ± 4.58 min) than in those who were treated by Z-scrotoplasty (45.50 min ± 6.67 min); this could recommend the utilization of Heineke-Mikulicz scrotoplasty in the treatment of such condition to save the anesthetic time.

The difference in the incidence of overall post-operative complications after using either Heineke-Mikulicz (HM) scrotoplasty or Z scrotoplasty was not significant however similar to the reported results of the previously described study comparing different techniques in the treatment of post-circumcision webbed penis [3].

The cosmetic results was acceptable in our study; this is similar to the finding of Negm MA et al. in their study who concluded favorable outcome in either techniques [3]. S. Chang et al. described a double V–Y advancement flaps at the penoscrotal region relieving the penoscrotal web without neither skin gangrene nor contractures and concluded that an acceptable outcome could be achieved by such technique if applied properly [4].

## Conclusion

The congenital webbed penis could be treated by either Heineke-Mikulicz scrotoplasty or Z scrotoplasty with satisfactory outcomes. The difference in surgical outcomes is not significant. The only significant factor is that Heineke-Mikulicz scrotoplasty takes a shorter operative duration.

### Study limitations

Further study is recommended based on degree-directed procedure in order to titrate whether there is a difference in the surgical outcome between degree directed procedures or not. A wider scale of patients have to be included in future study to compare the results of both techniques in different grades of penoscrotal web. Also, fixation of the penoscrotal web by fixation sutures could be added in the management of penoscrotal web in further studies.

## Data Availability

The datasets used and/or analyzed during the current study are available from the corresponding author on reasonable request.
